# Mindfulness and Emotion Regulation: Insights from Neurobiological, Psychological, and Clinical Studies

**DOI:** 10.3389/fpsyg.2017.00220

**Published:** 2017-03-06

**Authors:** Simón Guendelman, Sebastián Medeiros, Hagen Rampes

**Affiliations:** ^1^Social Cognition Group, Berlin School of Mind and Brain, Humboldt UniversitätBerlin, Germany; ^2^Research Unit on Psychotherapeutic Interventions and Change Processes, Millennium Institute for Research in Depression and PersonalitySantiago, Chile; ^3^Health Psychology, Department of Psychology, Pontificia Universidad Católica de ChileSantiago, Chile; ^4^Community Mental Health Team East, Central North West London Foundation NHS Foundation TrustLondon, UK

**Keywords:** mindfulness, emotion regulation, neuroimaging, top down and bottom up processing, emotion dysregulation disorders, embodied cognition

## Abstract

There is increasing interest in the beneficial clinical effects of mindfulness-based interventions (MBIs). Research has demonstrated their efficacy in a wide range of psychological conditions characterized by emotion dysregulation. Neuroimaging studies have evidenced functional and structural changes in a myriad of brain regions mainly involved in attention systems, emotion regulation, and self-referential processing. In this article we review studies on psychological and neurobiological correlates across different empirically derived models of research, including dispositional mindfulness, mindfulness induction, MBIs, and expert meditators in relation to emotion regulation. From the perspective of recent findings in the neuroscience of emotion regulation, we discuss the interplay of top-down and bottom-up emotion regulation mechanisms associated with different mindfulness models. From a phenomenological and cognitive perspective, authors have argued that mindfulness elicits a “mindful emotion regulation” strategy; however, from a clinical perspective, this construct has not been properly differentiated from other strategies and interventions within MBIs. In this context we propose the distinction between top-down and bottom-up mindfulness based emotion regulation strategies. Furthermore, we propose an *embodied emotion regulation* framework as a multilevel approach for understanding psychobiological changes due to mindfulness meditation regarding its effect on emotion regulation. Finally, based on clinical neuroscientific evidence on mindfulness, we open perspectives and dialogues regarding commonalities and differences between MBIs and other psychotherapeutic strategies for emotion regulation.

Increasing interest has emerged about the therapeutic effects of mindfulness meditation and its clinical applications. Several studies have shown positive results in fostering emotional mental health among clinical and healthy populations (Bohlmeijer et al., 2010; Fjorback et al., 2011; Gotink et al., 2015). Neurobiological studies indicate that this type of mental training may have an effect on the plasticity of brain structure and functioning (Tomasino et al., 2013; Fox et al., 2014). Some of the main neurocognitive mechanisms implicated in mindfulness meditation include attention control, emotion regulation, and self-awareness (Tang et al., 2015). In this article, we will focus on the relationship between mindfulness and emotion regulation, taking into account diverse psychological, clinical and neuroimaging evidence.

Unlike other reviews on the topic, this article does not focus on the problematic aspects involved in the operationalization and definition of mindfulness itself. Instead, the intention is to offer a comprehensive perspective linking different empirical models including mindfulness as a trait, mindfulness inductions, MBIs and mindfulness experts, and emotion regulation-related mechanisms including psychological and top-down/bottom-up brain systems. Moreover, we propose a preliminary framework for better understanding of emotion regulation changes due to mindfulness practice, tackling problematic aspects of the notion of “mindful emotion regulation” widely used in mindfulness clinical research, and complex involvement of top-down and bottom-up mechanisms in MBIs.

## Mindfulness, emotion regulation, and clinical applications

Contemporary psychology considers emotion regulation a central component of mental health, and its imbalances might underlie several mental disorders (Berenbaum et al., 2003; Mennin and Farach, 2007). Emotion regulation includes all of the conscious and non-conscious strategies we use to increase, to maintain or decrease one or more components of an emotional response (Gross, 1998). Originally, trying to bring together ideas from psychoanalysis and the field of stress and coping behaviors, Gross developed a *process* or *time model* of emotion regulation, in which emotions can be modulated in five different stages: selecting a situation, modifying a situation, deployment of attention, changing cognition (cognitive reappraisal), and modulating the experience, behavior or physiological response (Gross, 2001). Gross and John in a correlational study demonstrated that individual differences in the usage of these strategies (more cognitive reappraisal) were related to better emotional health, well-being and interpersonal functioning (Gross and John, 2003).

In line with this approach, Aldao et al. performed a meta-analytic review focused on how emotion regulation strategies, measured by self-report scales, vary across different psychopathological conditions. The main findings showed that *avoidance, rumination*, and *suppression* (as strategies) were each positively associated with anxiety, depression and eating disorders. *Problem-solving* was negatively associated with anxiety, depression and eating disorders. *Reappraisal* and *acceptance*-based strategies were negatively associated, but not significantly, with anxiety and depression (Aldao et al., 2010). Emotion dysregulation has been recognized as a core psychopathological factor in many other psychological disorders such as borderline personality disorder (BPD; Linehan, 1993; Schore, 2003), emotional trauma (Corrigan et al., 2011), attention deficit hyperactivity disorder (ADHD; Shaw et al., 2014), bipolar disorder (Van Rheenen et al., 2015), and anorexia and bulimia nervosa (Lavender et al., 2015). Emotion dysregulation has been demonstrated to mediate the link between child abuse/neglect and later depressive disorder (Crow et al., 2014), and also the link between cumulative adversity in lifetime and depressive symptoms (Abravanel and Sinha, 2015).

Taking into account how individual differences in emotion regulation strategies influence mental health, and the extensive role of emotion dysregulation in many psychopathological conditions, it is reasonable to believe that clinical interventions focused on emotion regulation/dysregulation might have substantial benefits for these psychological disorders. This argument is in line with several studies in which MBIs seem to be particularly effective in clinical and non-clinical conditions characterized by distress and negative emotions.

Mindfulness meditation has its origin in the Buddhist psychology tradition, more specifically in the texts known as *Satipatthana Sutra* (Analayo, 2003) and the *Abhidharma* (from Sanskrit, means higher teachings), a cycle of teachings concern about how the mind, including emotions and consciousness work (Trungpa, 2001; Analayo, 2003; Rapgay and Bystrisky, 2009). The word “mindfulness” corresponds to the translation of the original terms *smrti* (from Sanskrit) or *sati* (Pali), which captures the capacity to retain an object in the mind, but in a broad sense also implies being aware of and attentive to the present moment (Lutz et al., 2015). In clinical and research contexts, mindfulness as a specific type of meditation practice has been described as a “non-elaborative, non-judgmental awareness” of present-moment experience (Kabat-Zinn, 2005), a non-reactive awareness that emerges as a result of intentionally paying attention to present experience, and a capacity that can be trained through formal meditation practice. Several MBIs have been developed, including mindfulness meditation and other components, such as body awareness, yoga, and psychoeducation. These are group interventions, specially designed for targeting specific psychopathological substrates (like emotion dysregulation), in particular those related to psychiatric conditions (Shonin et al., 2013).

The mindfulness-based stress reduction (MBSR) program was developed by Jon Kabat-Zinn during the late seventies (Kabat-Zinn, 2005). Several revisions and meta-analyses have highlighted its robust benefits for healthy subjects, increasing well-being, and decreasing stress and negative emotions (Eberth and Sedlmeier, 2012). For clinical population, highlights the decrease in pain intensity, stress, and psychological complaints among patients suffering from diverse chronic pain/inflammatory diseases (Cramer et al., 2012; Lauche et al., 2013) and cancer (Ledesma and Kumano, 2009). Recently, a standardized review of meta-analysis of randomized controlled trials (RCTs) for MBSR and mindfulness-based cognitive therapy (MBCT) demonstrated a significant improvement in different domains (calculated as Cohen's *d* effect sizes): depressive symptoms (*d* = 0.37), anxiety (*d* = 0.49), stress (*d* = 0.51), quality of life (*d* = 0.39), physical functioning (*d* = 0.27; Gotink et al., 2015).

MBCT is a program derived from MBSR, developed for preventing recurrence/relapse in recurrent major depressive disorder (MDD; Segal et al., 2002). Several RCT and systematic reviews have demonstrated its effectiveness in relapse prevention and residual symptoms (Chiesa and Serretti, 2011; Piet and Hougaard, 2011; Clarke et al., 2015), and lately, also, in depressive symptoms in MDD (Jain et al., 2015). Another MBI is mindfulness-based relapse prevention (MBRP), which is designed for preventing relapse in substance use disorders (Bowen et al., 2010). Available studies have demonstrated its efficacy in reducing relapse into drug and drinking use, as well as substance usage after a period of abstinence (Bowen et al., 2014; for summary of results, see Table 1).

**Table 1:** **Summary of mindfulness-based interventions (MBIs) and main evidence-based targeted conditions**.

**MBI**	**Main conditions with evidence support for MBI**
MBSR	Stress, burnout (health professions)
	Chronic pain (low-back pain, fibromyalgia)
	Cancer
MBCT	MDD (relapse prevention and acute treatment). BD
MBRP	Substance use disorders (relapse prevention)
ACT	Chronic pain, anxiety and depressive disorders
DBT	Borderline personality disorder, substance use disorders

*MBSR, mindfulness based stress reduction; MBCT, mindfulness based cognitive therapy; MBRP, mindfulness based relapse prevention; ACT, acceptance and commitment therapy; DBT, dialectical behavioral therapy*.

Acceptance and commitment therapy (ACT) is a particular psychotherapeutic orientation developed from behavioral analysis, with mindfulness and acceptance as core principles (Hayes et al., 1999), whose effectiveness is similar to that of cognitive behavioral therapy (CBT) for relevant mental disorders (A-Tjak et al., 2015). Dialectical behavioral therapy (DBT) was developed within a CBT framework, and combines mindfulness and ACT elements. It is organized as a yearlong program, targeting self-harm, and chronic suicidal behavior in BPD (Linehan, 1993). Systematic reviews of ACT find decreases in impulsivity and suicidal attempts, and improvements in general mental health (Stoffers et al., 2012). Interestingly, for the MBIs clinical programs, the central aim is to target dysfunctional strategies of emotion regulation, which are claimed to drive the maintenance and recurrence of these disorders. In this sense, the claim is that mindfulness might re-establish emotion regulation capacities, which leads to symptomatic and clinical recovery.

## Psychological mechanisms of emotion regulation involved in mindfulness

Despite the effectiveness of MBIs in different psychological disorders, the underlying psychological and neurobiological mechanisms are still unclear. Several authors have proposed psychological models to account for the therapeutic effects of MBIs. Shapiro et al. claim that mindfulness might act through changing attention, intention, and attitude (Shapiro et al., 2006). Others suggest that positive effects of MBIs could be explained by mechanisms such as observing, describing, acting with awareness, non-judging of inner experiences, and non-reactivity to inner experiences (Baer et al., 2006). Based on an integration of Buddhist psychology and empirical evidence, Grabovac et al. proposed a model in which changes in acceptance, attention regulation, ethical practice, and attachment/aversion to feelings lead to decreased mental proliferation (rumination narrative based), and through this to salutary effects (Grabovac et al., 2011).

Other authors have proposed neurocognitive models, integrating psychological and neuroscientific data. Vago and Silbersweig proposed that mindfulness leads to changes in self-processing, through the development of self-awareness (meta-awareness), self-regulation (modulation of behavior), and self-transcendence (prosocial characteristics). These changes reflect modulation in neurocognitive networks related to intention and motivation, attention and emotion regulation, extinction and reconsolidation, prosociality, non-attachment, and decentering (Vago and Silbersweig, 2012). Hölzel et al. proposed that mindfulness enacts its effects through plastic changes of mental and brain functions related to attention regulation, body awareness, emotion regulation and self-perspectives (Hölzel et al., 2011a). Recently, Lutz et al. developed a multidimensional model for understanding mindfulness in expert meditators and MBIs, proposing a neurophenomenological “matrix model” in which categorical orthogonal dimensions, including object orientation, dereification and meta-awareness, are central cognitive mechanisms underlying contemplative practices (Lutz et al., 2015; for summary of models, see Table 2).

**Table 2:** **Psychological and neurocognitive models of mechanisms of MBIs**.

**Author**	**Type of model**	**Components**
Shapiro et al., 2006	Psychological	Attention, attitude, intention
Baer et al., 2006	Psychological	Observing, describing, acting with awareness, non-judging of inner experiences and non-reactivity to inner experiences
Grabovac et al., 2011	Psychological	Acceptance, attention regulation, ethical practice and decreased attachment/aversion to feelings. Final pathway: *decreased mental proliferation* (rumination narrative based)
Vago and Silbersweig, 2012	Psychological-Neurocognitive	Intention and motivation, attention and emotion regulation, extinction, and reconsolidation, prosociality, non-attachment, and decentering. Final pathway: *increasing self-awareness, self-regulation, self-trascendence*
Hölzel et al., 2011a	Psychological-Neurocognitive	Attention regulation, body awareness, emotion regulation, and change in perspective of the self. Final pathway: *increasing self-regulation*
Lutz et al., 2015	Phenomenological-Neurocognitive	Primary (orthogonal) dimensions: object orientation, dereification, and meta-awareness. Secondary qualities: aperture, clarity, stability, and effort

As can be seen, the nature and usage of the construct of mindfulness are complex and elusive. In order to understand the myriad of studies reviewed in this article, it's necessary to clarify the different usage of the mindfulness construct. *Dispositional mindfulness* is understood as a mental trait or stable characteristic of personality, which can vary between and within individuals across time. *Mindfulness as practice* refers to the concrete practice of mindfulness meditation, the deployment (and training) of a non-elaborative (non-conceptual), present-centered, exploratory and non-judgmental (non-valorative) awareness. *Mindfulness as a state* corresponds to the actual proper first-person experience of the non-elaborative, present-centered, non-judgmental awareness (Chambers et al., 2009; Davidson, 2010).

Although most of these models include cognitive, self-awareness, emotional, and attitudinal components, none of them provide an in-depth understanding of the relationship between mindfulness and emotion regulation changes. As can be derived from previous section, a lot of clinical evidence indicates that MBIs seem to be particularly effective in psychological conditions characterized by different forms of emotion dysregulation (see Table 1). In accordance with this, authors studying the psychological mechanisms underlying *mindfulness as a trait* or *as a practice* have focused specially on the relationship between mindfulness and its capacity to enhance emotion regulation as a key route to yielding mental health benefits.

### Cross-sectional studies

Studies measuring dispositional mindfulness consist of cross-sectional surveys using self-report scales in a healthy population. The frequency of these studies has grown exponentially and their scope has moved beyond psychiatry and psychology issues to include several other positive health-related outcomes. For example, recent studies suggest that higher dispositional mindfulness is correlated to improved self-care behaviors (Slonim et al., 2015), and among people with adverse childhood experiences, mindfulness as a trait is related to fewer medical conditions, and better health behaviors (Whitaker et al., 2014).

Giluk performed a meta-analysis of 29 studies investigating the relationship between mindfulness and personality (Big Five) and aspects of affect/mood, finding a negative correlation between mindfulness, neuroticism and negative affect, and a positive correlation between mindfulness and conscientiousness and positive affect (Giluk, 2009). Feltman et al., in a study with 289 participants, found that mindfulness and neuroticism were independent and inverse predictors of depressive symptoms and trait anger; importantly the relationship between neuroticism and symptoms was stronger with low mindfulness, suggesting that mindfulness might play a role in buffering the negative emotionality of neuroticism (Feltman et al., 2009). In line with this, Wupperman et al. found that deficits in mindfulness predict borderline symptoms in a healthy population, independently of neuroticism (Wupperman et al., 2008).

Other studies have evaluated what factors mediate the effect of mindfulness on emotion symptomatology. Bao et al. found a mediation effect of mindfulness, through increases in emotional intelligence (including factors such as emotion regulation) over perceived stress (Bao et al., 2015). Selby et al. looked at how borderline symptoms predict low mindfulness levels. Performing a bootstrapping mediation analysis revealed a significant effect of rumination as a mediator between borderline features and mindfulness deficits, indicating the maladaptive role of rumination as a regulatory strategy (Selby et al., 2016). These results are congruent with intervention studies that highlight the positive effect of DBT and ACT in the BPD population (Gratz and Gunderson, 2006; Stoffers et al., 2012).

Looking to further clarify and understand psychological mechanisms of mindfulness, Coffey et al. conducted a correlational study with 399 healthy people using the five-factor mindfulness questionnaire, the difficulties in emotion regulation scale and the trait meta-mood scale. Using factor analysis and structural equation modeling, the authors found that mindfulness and emotion regulation corresponded to shared and distinct constructs, distinguishing four factors: present-centered attention and acceptance of experience (for mindfulness), clarity about one's internal experience, and the ability to manage negative emotions (for emotion regulation). A path analysis supported the stance that mindfulness (including the factors “present-centered attention” and “acceptance of experience”), through clarity about one's own experience, improves the ability to deal with negative emotions (the model had a good data fit, having a RMSEA of 0.059; *p* < 0.0001). The authors also found that clarity about experience was negatively correlated to rumination and psychological distress, and positively related to flourishing (Coffey et al., 2010). Acknowledging methodological limitations, studies using dispositional mindfulness as a trait or personality characteristic (statistically as independent variable or predictor) provide interesting preliminary evidence that mindfulness, even though partially overlapping with emotion regulation constructs, might exert its beneficial salutary effects through higher emotion regulation capacities.

### Longitudinal studies

In the area of clinical and psychotherapy research, the question of change mechanisms, or “active ingredients,” that drive therapeutic effects has been a central concern over the last 20 years (Kazdin, 2007; Nock, 2007). As we stated in previous sections, hundreds of longitudinal studies have demonstrated the efficacy of MBIs in a healthy or clinical population, but also studies have evaluated change factors that might mediate the salutary effects of these interventions.

Recently, Gu et al. performed a systematic review and meta-analysis only of MBSR and MBCT studies that included mediation analysis. Starting from 169 trials and ending with 20 included in further analyses, the authors found consistent and strong evidence of emotional and cognitive reactivity, repetitive negative thinking (such as rumination and worry), and mindfulness itself as change factors/mechanisms. Only for mechanisms with sufficient studies (mindfulness and repetitive negative thinking) was quantitative synthesis using two-stage meta-analytic structural equation modeling used, further confirming mindfulness and rumination/worry as mediators of the effects of MBIs (Gu et al., 2015). In the same vein, intending to understand change mechanisms using MBCT for recurrent depressive disorder, Maj van der Velden et al. performed a systematic review of mediation studies. Out of 23 studies, 12 showed that mindfulness skills, worry, rumination, self-compassion and meta-awareness mediated or predicted treatment outcomes of MBCT (Van der Velden et al., 2015).

From these meta-analytic reviews, including high-quality RCT mediation studies, it is possible to state that mindfulness, emotional and cognitive reactivity, rumination/worry, self-compassion, and meta-awareness might be mechanisms underlying the therapeutic effects of MBIs (for summary of mechanisms, see Table 3). On the one hand, increases in *mindfulness, self-compassion*, and *meta-awareness* might account for adaptive emotion regulation strategies; on the other hand, decreases in emotional, cognitive reactivity, and rumination/worry might represent the dismantling of dysfunctional emotional-cognitive and self-processing strategies of emotion regulation. This evidence is concordant with the work of Aldao et al. in which *avoidance, rumination*, and *suppression* as emotion regulation strategies were correlated to anxiety, depression, and eating disorders (Aldao et al., 2010). Therefore, MBIs might target specific emotion regulation deficits of emotion-related disorders.

**Table 3:** **Evidence-based putative psychological mechanisms of MBIs (MBSR/MBCT)**.

**Author**	**Emotional**	**Cognitive**	**Attitudinal**
Gu et al., 2015	<Emotional reactivity	<Cognitive reactivity	>Mindfulness
		<Rumination	
		<Worry	
Van der Velden et al., 2015	>Self-compassion	>Meta-awareness	>Mindfulness
		<Worry	
		<Rumination	

## Neural mechanisms of emotion regulation involved in mindfulness

As we have stated before, emotion regulation can be defined as all the conscious and non-conscious strategies we use to increase, maintain or decrease one or more components of an emotional response (Gross, 2001), including *implicit*, non-conscious, and automatic processes, as well as *explicit*, voluntary and conscious mental processes (Gyurak et al., 2011). From a neural perspective, these processes are realized by different and complex distributed brain systems. Subcortical regions like the amygdala, periaqueductal gray, ventral striatum (VS), anterior insula (AI), and dorsal-anterior cingulate cortex (dACC) are involved in *emotional reactivity*, as emotion generation regions leading changes in arousal and valence regarding the triggering stimuli. Cortical regions such as the dorso-lateral prefrontral cortex (dLPFC), the ventro-lateral prefrontral cortex (vLPFC), the pre-supplementary and supplementary motor area (pre-SMA and SMA) and parietal cortex are involved in *explicit emotion regulation*. These regions conform to the so-called *central executive network* (CEN), usually involved in top-down emotion regulation, but also in attention and voluntary cognitive control. Finally, the ventral-anterior cingulate cortex (vACC) and the ventro-medial prefrontal cortex (vMPFC) are involved in *implicit emotion regulation*, the outside of awareness processing of emotion, but also in encoding subjective value of the stimuli or condition experienced by the subject (Frank et al., 2014; Kohn et al., 2014; Etkin et al., 2015). From now on, we will refer to the explicit emotion regulation system as the top-down system, and to the emotion generation and the implicit emotion regulation systems as both part of a bottom-up system, since both feed up the top-down system with information regarding arousal, visceral homeostasis, aversiveness and rewardingness of a given stimuli or situation, among others.

It has been stated that different emotion regulation strategies might differentially activate these brain systems implicated in emotion regulation processes. For example, Dörfel et al. found that *detachment, distraction (two forms of reappraisal), and expressive suppression* increase brain activation in the same regions of the right fronto-parietal network, reducing activation of the left amygdala. This suggests a common underlying neural process for these strategies, but somewhat contrary to theoretical predictions, since *expressive suppression* as a less adaptive strategy might have a different neural correlate from reappraisal strategies. Interestingly, only *reinterpretation* induced a different activation pattern, recruiting the left vLPFC and orbitofrontal gyrus, but not decreasing amygdala activation (Dörfel et al., 2014). In another study comparing *reappraisal* and *affect labeling*, authors found a common activation pattern including activation in the right and left dLPFC, right and left vLPFC, and pre-SMA, and decreased amygdala and vMPFC activation (Burklund et al., 2014). Recently, a meta-analysis of 48 studies of cognitive reappraisal emotion regulation neuroimaging studies concluded that this strategy particularly activates the bilateral dLPFC, vLPFC, dMPFC, posterior parietal cortex, and left-middle temporal gyrus, and deactivates the amygdala bilaterally. Clearly involving the explicit emotion regulation network. Unexpectedly, no other regions related to emotion reactivity decreased their activation level during reappraisal down regulation (Buhle et al., 2014).

Interestingly, some studies have demonstrated that the top-down or explicit emotion regulation system (dLPFC, vLPFC, parietal cortex) can also be involved in generating emotional states and not only in controlling them, in conjunction or in parallel with the implicit emotion generation system (Ochsner et al., 2009; McRae et al., 2012). In particular, in two studies, applying cognitive reappraisal to emotions generated via implicit stimulation resulted in a paradoxical increased activation of the amygdala (Herwig et al., 2010; McRae et al., 2012). In Herwig et al.'s study, the usage of emotional body-awareness strategy decreased amygdala activation compared to reappraisal strategy (Herwig et al., 2010). These studies highlight the question of whether top-down emotion regulation strategies are always the most appropriate, and whether there are other effective forms of emotion regulation that are not based on top-down mechanism.

Of particular interest for the mindfulness-based emotion regulation field is the notion of bottom-up emotion regulation. At the brain mechanisms level, the main assumption of this model is that the bottom-up systems implying emotional generation regions (like the amygdala, dACC and AI) and implicit emotion regulation regions (like the vMPFC) can also be modulated without the involvement of cognitive control (like the v-d LPFC), or semantic processing regions (temporal cortex). Several authors have argued that mindfulness might exert a unique emotion regulation strategy, termed “mindful emotion regulation,” different from cognitive reappraisal (based on top-down system), mainly through the privileged engagement of these bottom-up emotion regulation systems (Chambers et al., 2009; Farb and Segal, 2012; Chiesa et al., 2013; Grecucci et al., 2015a). Nevertheless, whether mindfulness-based emotion regulation is a unique phenomena, and whether it only relies on the involvement of bottom-up systems excluding cognitive control regions (top-down systems), and what the exact brain signature of mindfulness is as an emotion regulation strategy, among other questions, are still a matter of debate and will be addressed in the following sections of the article.

### Structural brain changes in mindfulness experts and mindfulness-based interventions

Several studies have investigated the effect of MBIs and long-term mindfulness meditation practice using structural brain imaging, like morphometry-based magnetic resonance imaging (MRI) techniques. Cross-sectional design studies comparing healthy controls with expert meditators (EMs) from different meditation traditions have demonstrated structural MRI changes in: the hippocampus (Hölzel et al., 2008; Luders et al., 2009; Kang et al., 2013); right anterior insula (AI; Lazar et al., 2005; Hölzel et al., 2008); orbitofrontal cortex (OFC; Hölzel et al., 2008; Luders et al., 2009; Kang et al., 2013); anterior cingulate cortex (ACC; Grant et al., 2013); left temporal pole (TP; Hölzel et al., 2008; Luders et al., 2009; Kang et al., 2013); left frontal gyrus (Vestergaard-Poulsen et al., 2009; Kang et al., 2013); right frontal sulcus (Lazar et al., 2005); corpus callosum (Luders et al., 2012; Kang et al., 2013); and regions in the brainstem (Vestergaard-Poulsen et al., 2009). Moreover, a study using machine learning structural pattern recognition analysis estimated that brains of meditators were 7.5 years younger than matched control subjects (Luders et al., 2016).

As can be seen, covering a wide range of brain regions, according to recent reviews and meta-analysis of neural bases of emotion regulation (Frank et al., 2014; Kohn et al., 2014; Etkin et al., 2015), would partially overlap with emotion reactivity (AI, ACC), and with implicit emotion regulation regions (OFC and vMPFC), and very loosely with explicit emotion regulation (medial PFC, but not lateral PFC regions) systems. From this, if mindfulness meditation would involve cognitive reappraisal, or top-down emotion regulation strategies, one would expect changes in lateral PFC morphometry. It is important to note that due to the design of the studies, it is not possible to infer causality between brain changes and long-term meditation practice; also, because of the nature of brain structural imaging, it is not possible to derive any information about brain regions' functions. Another limitation of these studies is the variability of hours of meditation practice within this population, ranging from 1,000 to 10,000 or more hours. Nevertheless, they might offer preliminary evidence of the effects of long-term mindfulness practice on brain plasticity.

During the last few years, longitudinal studies have assessed the impact of MBIs on brain morphology, particularly the MBSR 8-week program. Hölzel et al., using MRI voxel-based morphometry (VBM), found changes in gray matter density in the left hippocampus, posterior cingulate cortex, right temporo-parietal junction (TPJ), some small regions in the brainstem, and cerebellum (Hölzel et al., 2011b). In a similar uncontrolled longitudinal study with MBSR, the authors found that decreases in perceived stress were correlated to a decreased gray matter density in the right amygdala (Hölzel et al., 2009). They also found a correlation between major psychological well-being and plastic changes in the brainstem (Singleton et al., 2014). Santarnecchi et al. performed a controlled longitudinal study with MBSR, finding a significant increase in cortical thickness in two clusters: the right SSC and right paracentral lobule, and AI and right inferior frontal gyrus (operculum). The authors found a significant interaction between structural changes in the right insula and a decrease in alexithymia levels, suggesting “body or interoceptive awareness” as a possible mechanism responsible for salutary effects of mindfulness practice (Santarnecchi et al., 2014).

These studies suggest that an 8-week MBI (MBSR) might induce neuroplastic changes in key areas for emotional reactivity (amygdala, insula), body awareness or interoception/exteroception (insula, somatosensory cortex), self-consciousness (posterior cingulate cortex, pons), mood, and arousal regulation (brainstem regions—locus coeruleus, and raphe nuclei), perspective taking (TPJ) and memory systems (hippocampus, cerebellum). Interestingly, none of these studies suggest changes in PFC areas or regions involved in the top-down emotion regulation system, thereby indicating that salutary effects of MBI might be mediated mainly by changes in particular relevant subcortical and cortical regions related to bottom-up or non-emotion regulation related functional systems.

### Functional brain changes in emotion tasks in mindfulness studies

#### Dispositional mindfulness

Cross-sectional studies in healthy populations have investigated how individual differences in mindfulness as trait might be related to specific brain functions during emotion elicitation task experiments. Creswell et al., in an affect labeling task during fMRI, found that levels of dispositional mindfulness were related to higher activations in the right vMPFC and right vLPFC and major deactivation of the right amygdala (Creswell et al., 2007). In a similar study, participants were asked to observe emotional faces during fMRI, and higher levels of DM were correlated to less amygdala reactivity. Using resting-state functional connectivity (rs-fMRI) analysis, the authors found a relationship between higher dispositional mindfulness and decreased connectivity within the midline regions, including the PCC and MPFC (Way et al., 2010). Importantly, the midline regions like the MPRC, PCC, precuneus, ACC, and parietal cortex are part of the so-called default mode network (DMN; Raichle and Snyder, 2007), which has been related to mind-wandering (task-unrelated thought) and self-referential processing (Qin and and Northoff, 2011). Brown et al. assessed 46 participants with an electro-encephalogram (EEG) while viewing emotionally laden pictures, particularly looking at the late positive potential (LPP) as a marker of affective processing. Authors found that higher dispositional mindfulness correlated to lower LPP during high-arousal negative images (Brown et al., 2013). Finally, Kong et al., using rs-fMRI and local synchronization measurements (estimated by regional homogeneity) with 290 subjects, found that major dispositional mindfulness correlated to local synchronization in the right insula, left OFC, left parahippocampal gyrus (regions involving emotion reactivity, implicit emotion regulation), and decreased local synchronization with the inferior frontal gyrus (IFG; related to explicit emotion-regulation). Furthermore, levels of local synchronization in the OFC predicted positive emotions, and in the IFG predicted a sense of meaning and purpose in life, both effects mediated by DM (Kong et al., 2016). This study suggests that local synchronization in key regions of emotion regulation might engage differently in subjects high in dispositional mindfulness, accounting for positive emotions' salutary effects. Also it shows no correlation between lateral PFC local synchrony and dispositional mindfulness in emotion regulation-related variables, suggesting that individuals high in dispositional mindfulness might engage in emotion-related processes involving different regulatory systems than top-down ones (for summary of results, see Table 4).

**Table 4:** **Summary of neuroimaging studies using emotion-task experiments in different mindfulness conditions**.

**Mindfulness condition (different models) vs control condition (Waiting list, or active control)**	**Study design**	**Population**	**Sample size: mindfulness (M) vs control groups (C)**	**Experimental task and neuro-imaging method: resting state functional magnetic resonance imaging (rs-fMRI), task based functional magnetic resonance imaging (fMRI), electroencephalography (EEG)**	**Main finding: summarized in terms of brain, and/or physiological response changes**	**References**
**DISPOSITIONAL MINDFULNESS**						
Dispositional mindfulness	Cross sectional/Uncontrolled study	Healthy	M: 27	Affect labeling task during fMRI	Level of DM mediates the relationship between right vMPFC, right vLPFC activation and right amygdala deactivation	Creswell et al., 2007
Dispositional mindfulness	Cross sectional/Uncontrolled study	Healthy	M: 27	Viewing negative emotional faces during fMRI + rs-fMRI	Higher DM correlated with less amygdala reactivity. Also with less resting connectivity in midline brain regions (self-referential processing)	Way et al., 2010
Dispositional mindfulness	Cross sectional/Uncontrolled study	Healthy	M: 46	Viewing negative/positive pictures during EEG (LPP: late positive potential)	Higher DM correlated to lower LPP during high-arousal negative emotions	Brown et al., 2013
Dispositional mindfulness	Cross sectional/Uncontrolled study	Healthy	M: 290	rs-fMRI—local synchronization	Higher DM correlated to local synch in left OFC, left parahippocampal gyrus, right insula. Local synch in OFC-predicted positive affect, and in IFG-predicted purpose/meaningful life	Kong et al., 2016
**MINDFULNESS INDUCTION**						
Mindfulness induction	Cross sectional/Uncontrolled study	Smokers looking for treatment to stop smoking	M: 47	Cue-induced craving during fMRI.	Reduced neural activity in sg-ACC [craving-related—emotion reactivity region] and a reduced functional connectivity between this same region with the bilateral insula and ventral striatum with no direct involvement of PFC regions (^*^)	Westbrook et al., 2013
Mindfulness induction	Cross sectional/Non-randomized controlled study	Healthy	M: 24/C: 22	Cued expectation and perception of negative pictures during fMRI	During expectation major activations in prefrontal regions: left AI, right and left dMPFC and left dLPFC. During perception reduced activation in right amygdala and parahippocampal gyrus [emotion processing reactivity] (^*^)	Lutz A. et al., 2013
Mindfulness induction *vs* Reappraisal strategy	Cross sectional/Non-randomized controlled study	Healthy	M: 24/C: 23	Cued expectation and perception of negative pictures during fMRI	Both groups: similar activity of the m-PFC and the amygdala. Major activations in MI group, during expectation: vLPFC, vLPFC, Supramarginal gyrus and left insula. During perception: major activity in the caudate in the cognitive group	Opialla et al., 2014
**MINDFULNESS-BASED INTERVENTIONS**						
Mindfulness-based stress reduction	Cross sectional/Non-randomized controlled study (novice vs those who attended the course)	Healthy	M: 20/C: 16	Self-reference task during fMRI	Significant difference in the neural correlates of the self-reference task, during experiential focus an increased activation in right brain regions: lateral PFC, insula, second somatosensory area, and IPL	Farb et al., 2007
Mindfulness-based stress reduction *vs* Waiting list	Longitudinal/Non-randomized controlled study	Healthy	M: 20/C: 16	Sadness induction paradigm during fMRI	MBI group changed activation pattern in key emotion regulation regions: major activation in the right anterior insula, r-lPFC and sg—ACC.	Farb et al., 2010
Mindfulness-based stress reduction	Longitudinal/Non-Controlled trial	Social Phobia	M: 16	Breath focus task during fMRI	Reduced amygdala activity, major activation in precuneus, SPL, IPL compared to distraction focus task	Goldin and Gross, 2010
Mindfulness Training (4 days)	Longitudinal/Non-Controlled trial	Healthy (pain)	M: 15	Breath focus meditation during noxious stimulation task in fMRI	MBI reduction in pain intensity: major activation in ACC, anterior insula. MBI reduction in pain unpleasantness: major activation in OFC and thalamus (^*^)	Zeidan et al., 2011
Mindfulness-based stress reduction *vs* Aerobic exercise	Longitudinal/Randomized controlled trial	Social Phobia	M: 31/C: 25	Self-reference task during fMRI	MBIs during negative self-view: major activation in PCC, and dMPFC activity-associated less social anxiety disability and mindfulness level	Goldin et al., 2012
Mindfulness-based stress reduction *vs* Aerobic exercise	Longitudinal/Randomized controlled trial	Social Phobia	M: 31/C: 25	Emotion regulation of negative self-beliefs task during fMRI	MBI regulating negative self-beliefs: fewer negative emotions, major activation in R-IPL, R-SPL	Goldin et al., 2013
Mindfulness-based stress reduction *vs* Stress management education	Longitudinal/Randomized controlled trial	Generalized Anxiety Disorder	M: 15/C: 11	Affect labeling of emotional expressions during fMRI	Both groups less amygdala activation. MBI major activation in vLPFC. Increase functional connectivity between amygdala and PFC regions	Hölzel et al., 2013
Mindfulness Training (6 weeks) *vs* Shared reading and listening group	Longitudinal/Randomized controlled trial	Healthy	M: 30/C: 31	Affective Stroop conflict resolution task during fMRI	Both groups improved significantly in a response inhibition task. MBI reduced emotional interference, in negative emotion processing: increased bilateral dLPFC, right anterior insula and m-PFC (^*^)	Allen et al., 2012
Mindfulness Training (8 weeks) *vs* Compassion training *vs* Health discussion group	Longitudinal/Randomized controlled trial	Healthy	M: 12/C: 12/Compassion Training: 12	Observation of emotional pictures during fMRI	In MBI: decrease in right amygdala activation (all valences). In Compassion Training: trend increase in right amygdala response in negative pictures (^*^)	Desbordes et al., 2012
Mindfulness Training (4 days) *vs* Sham mindfulness *vs* Placebo *vs* Control	Longitudinal/Randomized controlled trial (four-arm)	Healthy (pain)	M: 80	Pain regulation strategy during noxious stimulation task in fMRI	MBI reduction in pain intensity: major activation in sg—ACC, anterior insula, OFC. Placebo analgesia: major activation in DLPFC and secondary somatosensory cortex (^*^)	Zeidan et al., 2015
**EXPERT MEDITATORS**						
Tibetan Buddhist monks	Cross sectional/Case-control study	Healthy	M: 14/C: 16	Auditory stimuli during focus attention task in fMRI	EM: amygdala deactivation	Brefczynski-Lewis et al., 2007
Tibetan Buddhist monks	Cross sectional/Case-control study	Healthy	M: 15/C: 15	Auditory stimuli during active compassion meditation in fMRI	EM: increased activation in the anterior insula and ACC, proportional to compassion experience intensity	Lutz et al., 2008a
Zen Western *vs* novices meditators	Cross sectional/Case-control study	Healthy	M: 12/C: 8	Observation of emotional pictures during active meditation in fMRI	EM during meditation: major deactivation of m-PFC and PCC. Relative deactivation of amygdala and insula vs novice meditators. Novice during meditation: downregulation of amygdala	Taylor et al., 2011
Zen Western	Cross sectional/Case-control study	Healthy	M: 13/C: 13	Noxious stimulus during fMRI	EM during pain: reduced activation in PFC, amygdala, hippocampus. Major activations in ACC, insula, thalamus.	Grant et al., 2011
Vipassana	Cross sectional/Case-control study	Healthy	M: 17/C: 17	Noxious stimulus during fMRI	EM during pain in meditation: reduced activation in lateral PFC, major activation in ACC, R-posterior insula	Gard et al., 2012
Tibetan tradition	Cross sectional/Case-control study	Healthy	M: 14/C: 14	Noxious stimulus during fMRI	EM: equal pain, less unpleasantness. During pain: major AI, ACC. Minor baseline activation AI, ACC, amygdala	Lutz A. et al., 2013
Buddhist Western	Cross sectional/Case-control study	Healthy	M: 18/C: 26	Dictator Game (DG) and Ultimatum Game (UG) during Skin Conductance Level (SCL)	EM: in DG reduced arousal, distress and SCL. In UG accept more unfair offers	Grecucci et al., 2015b
Buddhist Western	Cross sectional/Case-control study	Healthy	M: 26/C: 40	Ultimatum game during fMRI	EM: in UG accept more unfair offers. Major activation of the posterior insula (interoception) versus anterior insula (emotion reactivity) in controls; major activation in somatosensory and posterior superior temporal cortex	Kirk et al., 2011
Zen Western	Cross sectional/Case-control study	Healthy	M: 34/C: 44	Monetary incentive delay during fMRI	EM during reward anticipation: reduced activation in caudate nucleus, major activation in bilateral posterior insula. During reward receipt: reduced activation in vMPFC	Kirk et al., 2011
Buddhist Western	Cross sectional/Case-control study	Healthy	M: 28/C: 30	Passive conditioning task during fMRI	EM during reward prediction: reduced positive and negative prediction error BOLD in putamen. Major activation in posterior insula	Kirk et al., 2015

* *Finding indicates bottom-up mechanisms*.

Interestingly, these findings are concordant with psychological studies linking dispositional mindfulness to better emotional life outcomes (positive affect and emotional intelligence and minor neuroticism, negative affect, rumination, and borderline symptoms) thereby providing preliminary support for the construct validity of DM. These studies face many limitations, such as the difficulty in deriving causal inferences, and disentangling relevant confounders such as psychological traits and biological differences. Another problematic claim of these studies is the assumption that dispositional mindfulness really reflects daily-life mindful attitudes. At this time, to the best of our knowledge, no study has empirically clarified this point.

#### Mindfulness inductions

Studies using brief meditation practice, or mindfulness inductions, have started to explore the clinical utility (effectiveness) and neural underpinnings of these types of interventions. Westbrook et al. performed a cross-sectional study with smokers looking to stop smoking. Participants were asked to watch specific craving-inducing images during fMRI, using “mindful attention” vs. “passive viewing” as strategies. When applying “mindful attention,” subjects reported less craving impulse; additionally, they presented decreased activation in the subgenual ACC (sg-ACC), and reduced functional connectivity between this same region and bilateral AI and VS. At the same time, no involvement of the PFC was detected (Westbrook et al., 2013). Interestingly, sg-ACC, AI, and VS correspond to emotion generation regions, but are also implicated in other relevant affective functions such as craving and reward processing (VS), processing of salient stimuli and interoception (AI), and the subjective encoding of value and processing of emotional conflict (sgACC; Wilcox et al., 2016).

Lutz et al., in a cross-sectional study with healthy participants, compared one group applying mindfulness with a no-strategy group while looking at a set of emotional pictures during fMRI. When expecting negative pictures, the mindfulness group displayed increased activation of the left AI, right and left dMPFC, and left dLPFC. During perception of negative pictures, the mindfulness group showed reduced activation in the right amygdala and parahippocampal gyrus, with no involvement of the PFC (Lutz J. et al., 2013). The same researchers also compared groups using mindfulness vs. cognitive reappraisal using the same emotional task as in fMRI. During the expectation of negative pictures, both groups showed a similar pattern of activation of the MPFC and the amygdala, and during the perception of negative images, decreased activation of the head of the right caudate in the mindfulness group was the only difference (Opialla et al., 2014). Interestingly, the first experiment comparing mindfulness vs. baseline conditions suggests a bottom-up (targeting emotion reactivity regions, with no changes in PFC) mechanism of mindfulness as emotion regulation strategy; instead, when adding an active regulatory strategy as comparison, it is almost impossible to differentiate at the neural level between the two emotion regulation strategies. However, the observed deactivation of the right caudate head might index decreased engagement of automated cognitive and motor responses (Parent and Hazrati, 1995), which might be linked to decreased automatic cognitive reactivity, known as a mindfulness mechanism (Gu et al., 2015).

Interestingly, this draws attention to the fact that even a short mindfulness induction, in people naive about meditation, can induce a distinguishable bottom-up brain activation pattern when comparing mindfulness as a strategy to baseline or no-strategy condition. Nevertheless, when compared to cognitive reappraisal, differences seem to vanish. This suggests that mindfulness meditation in naive practitioners is performed with the engagement of widespread brain regions including top-down and bottom-up regulatory systems. From the clinical perspective, these studies provide a valuable outlook for understanding neurobiological substrates of brief meditation practices, which are central components of many MBIs, like MBCT, ACT, or DBT, that intend to elicit “mindfulness states” to face difficult emotions and emotion dysregulation states.

As previously stated, these studies share limitations with cross-sectional design studies. These investigations raise particularly relevant problems in the discussion of mindfulness and emotion regulation mechanisms, starting with the question of the acquisition of the so-called mindfulness emotion regulation strategies—in other words, when and how a person *acquires* the capacity to elicit a “mindfulness state,” different from other mental states. And also, when and how a person *acquires* the capacity to use mindfulness as an emotion regulation strategy. Finally, the question of how this learning process can be distinctly measured from behavioral and brain signatures. These are central questions that future studies need to unravel.

#### Mindfulness-based interventions: longitudinal studies on emotion, pain, and anxiety

Over the last few years, longitudinal studies using fMRI have used a myriad of experimental tasks investigating emotion regulation changes secondary to MBIs. Farb et al. studied the impact of MBSR using fMRI under a sadness induction paradigm. After the intervention, the mindfulness group changed the activation pattern in key diverse emotion regulation regions: comparatively increased activation in the right AI, right LPFC and sg-ACC. The control group showed major activation in the left PFC, left superior temporal sulcus (STS), precuneus, and PCC, areas usually involved in self-awareness and semantic processing (Farb et al., 2010). From the same lab, using a self-referential task (self-narrative vs. self-experiential) during fMRI, an increased activation was found in similar right brain regions, LPFC, AI, second SSC and inferior parietal lobule (IPL), for the *self-experiential* focus. Conversely, a *self-narrative* focus engaged major activation in the left vMPFC, dMPFC, and PCC, all midline regions that mainly correspond with the DMN (Farb et al., 2007). These studies indicate a different engagement of brain regions during emotion regulation; although both groups displayed top-down mechanisms linked to explicit emotion regulation systems (right or left LPFC), only the MBI groups employed regions related to emotion reactivity (AI, ACC), interoception (AI) and somatosensory awareness (SSC, IPL).

Attempting to unravel the involvement of different emotion regulation systems implicated in mindfulness meditation, Allen et al. performed an RCT comparing a 6-week mindfulness training and an active control (sharing and listening training). Despite both groups improving significantly in a response inhibition task, only the MBI group showed reduced emotional interference under an affective Stroop conflict resolution paradigm (a task known to activate implicit emotion regulation processes). The authors found no differences between groups in behavioral and neural activations during negative affect processing. Nevertheless, the greater amount of mindfulness practice predicted increased activation of bilateral dACC, right AI, and MPFC during implicit negative emotional processing, suggesting both implicit and explicit emotion regulation plasticity as mechanisms underlying mindfulness training (Allen et al., 2012). Another RCT study compared the effects of an 8-week Mindful Attention Training (MAT) vs. Cognitively Based Compassion Training (CBCT) vs. active control while participants passively viewed affective pictures during fMRI. In a region of interest analysis, the authors found decreased activation in the right amygdala in the MAT group in response to images of all valences. Interestingly, a trend increase in activation of the right amygdala when viewing negative images in the CBCT group was found, and the extent of this increase was significantly correlated to reductions in depressive symptoms (Desbordes et al., 2012). Although not conclusive, both RCT studies provide evidence that MBIs might exert their effects on the level of emotion reactivity and implicit emotion regulation.

Other studies have evaluated the impact of MBIs on pain processing. Zeidan et al. performed a longitudinal uncontrolled study with 4-day MBI training, using Artetial Spin Labeling (ASL), a technique for estimating cerebral blood flow with MRI across time points. After the intervention, during a breathing meditation task, the authors found decreased perfusion of the MPFC and PCC (DMN), and a major activation of the AI, ACC, pre-SMA, OFC, VS, SSC, and posterior insula (PI). During a pain induction paradigm, minor activation of the contra-lateral SSC and increased activation in the ACC, AI, PI, and fronto-parietal operculum were reported. It is worthy of note that participants reported a significant decrease in pain intensity and unpleasantness (Zeidan et al., 2011). Later, the same authors performed a four-arm RCT comparing MBI vs. placebo vs. sham mindfulness using a pain induction paradigm with ASL MRI. Interestingly, all groups showed a significant reduction in pain intensity and unpleasantness, but the MBI demonstrated a unique brain mechanism including greater activation of the OFC, sg-ACC, and AI. In line with previous evidence, these studies highlight emotion reactivity (AI, ACC, VS) and implicit emotion regulation (OFC, vMPFC) systems as the main emotion regulation targets of MBIs, again notably without any major involvement of PFC-related systems (top-down emotion regulation).

Other researchers have explored the effects of MBIs in clinical populations. In one of the first such studies, Goldin and Gross conducted an MBSR longitudinal study with people suffering from social anxiety disorder (SAD). Comparing two emotion regulation strategies using an anxiogenic task with negative self-beliefs, the authors found that being breathing-focused (vs. distraction-focused) produced minor negative emotional experiences, decreases in amygdala activation, and increased activation of the PCC, SPL, and IPL (areas involved in top-down emotion regulation, but also in self-awareness and attention processing; Goldin and Gross, 2010). The same authors performed an RCT comparing MBSR with aerobic exercise (AE), also in SAD patients, in this case comparing mindful attention (metacognitive perspective of mental content) and reacting (thinking according to negative self-beliefs) as strategies for dealing with negative-self-belief-induced emotions. During the task, the MBSR group reported fewer negative emotions, and showed differential engagement of attention regulation areas, with increased activation of the right IPL and SPL, and decreased activation of the culmen and left lingual gyrus (Goldin et al., 2013), areas involved in the orienting-attention network, implicated in early spatial detection of stimuli (Posner et al., 2006). The authors interpreted this finding as suggesting that MBIs enhance approaching behavior/attention toward anxiogenic stimuli, a core deficit in SAD (Goldin et al., 2013). In the context of the same trial condition, both groups significantly decreased social anxiety symptoms, disability and negative self-attribution, while also increasing positive self-views. Examining the neural correlate of self-views, the MBSR group displayed larger responses in the PCC, and dMPFC, which correlated with minor social anxiety, disability, and increased mindfulness (Goldin et al., 2012). Finally, Hölzel et al. ran an RCT with generalized anxiety disorder (GAD) patients, comparing MBSR and psychoeducation treatment groups performing an emotion labeling task during fMRI. The findings highlighted small increases in amygdala activation in both groups, and major increases of activity in the vLPFC, as well as increased functional connectivity between these regions (Hölzel et al., 2013). These studies point toward the idea that MBIs target basic cognitive processes broadly involved in attention regulation, including information updating, response inhibition, and goal maintenance (Malinowski, 2013). Interestingly, these are core functions for the CEN, and for the top-down emotion regulation system (Okon-Singer et al., 2015). In sum, these studies provide evidence that MBIs might exert their effects through top-down/cognitive-control emotion regulation mechanisms. Besides sample size, noteworthy limitations of these studies include the lack of control of basal cognitive deficits in patients, and of personality and comorbidity factors, which might influence basal neuroimaging results.

#### Expert meditators (EMs): cross-sectional studies on emotion, pain, and reward

Lutz et al. used an annoying auditory task during fMRI, comparing Tibetan monks and controls during active compassion meditation. They found increased activity in the AI and ACC, which were proportional to first-person experience of compassion intensity (Lutz et al., 2008a). Using the same experimental task, but during focused-attention meditation, researchers also found a direct relationship between meditation expertise (total hours of practice) and amygdala deactivation (Brefczynski-Lewis et al., 2007). Taylor et al. compared Western EMs with novel meditators using emotional pictures during fMRI, and observed a decrease in activation levels of the PCC and MPFC (DMN) during active meditation in EMs. During passive observation, beginner meditators showed major amygdala activation increases for negative affective pictures (Taylor et al., 2011). These studies highlight a specific modulation of the emotion generation system in EMs during emotion tasks.

Other studies have explored the effects of EMs in pain processing. Gard et al. compared Western EMs with controls, contrasting active meditation, and resting state using a pain induction paradigm with fMRI. The authors found no differences between groups in pain intensity, but in active meditation during pain induction, EMs referred less unpleasantness and a major activation in the right AI and a deactivation in the right and left inferior PFC (Gard et al., 2012). Grant et al. also compared EMs with controls during a pain induction task in fMRI. EMs showed decreased activation of the PFC, amygdala and hippocampus, and increased activity in the AI, ACC, and thalamus. Interestingly, the decreased functional connectivity between PFC and AI and ACC predicted lower pain in EMs (Grant et al., 2011). In a similar study, EMs showed lower baseline activation in the AI, ACC, and amygdala, and during pain induction higher activation of AI and ACC regions than controls (Lutz A. et al., 2013). These studies indicate that EMs specifically increases activation of subcortical emotion generation regions, related to affective processing of pain, and deactivates top-down mechanisms, evidencing a unique emotion regulation bottom-up mechanism.

Other studies have used reward or economic behavioral paradigms for studying emotion processing in EMs. Grecucci et al. compared EMs with a control group contrasting a “cognitive” vs. an “experiential” emotion regulation strategy during two monetary distribution tasks. While receiving offers in the dictator game, EMs showed decreased emotion arousal and physiological reactivity, with no effect of the strategy observed. While receiving unfair offers in the ultimatum game (UG), EMs accepted more unfair offers and performed less punishment, particularly during the “experiential” emotion regulation strategy (Grecucci et al., 2015a). Another study used fMRI during the execution of the UG. Compared to controls, EMs accepted more unfair offers, and during that particular condition engaged a particular functional brain response with greater activation of the PI than the AI, and major activation in the SSC and posterior superior temporal cortex (Kirk et al., 2011). Note that the PI is preferentially involved in interoception and the AI in emotion reactivity/generation and emotional awareness (Craig, 2009; Gu et al., 2013). These studies show that during socially induced negative emotions, EMs showed stronger modulation of their interactive behavior (less punishment) and greater emotion regulation, which was mediated via increased activation of interoception and exteroception brain regions, modulating emotion generation regions.

Kirk et al. used the monetary incentive delay task in EMs during fMRI, looking to disentangle the neural differences between anticipation and receipt of monetary reward. Compared to controls, during the anticipation phase EMs displayed decreased activation of the bilateral caudate, and increased activation of the bilateral PI. During the encoding of gains of reward, a minor activation of the vMPFC was seen (Kirk et al., 2015), indicating a dampening of the reward system. The same authors used a passive conditioning task (pairing a yellow light to juice intake) to evaluate how changes in the predictability of reward, encoded by the prediction error (PR) neural signal, differ between EMs and matched controls. In this task, the delay of the reward decreases PE (negative PE), while the intake of unexpected reward generates an increase in PE signal (positive PE). EMs were found to be less prone to positive and negative PE signals in the putamen (part of the striatum and the reward system), which again was associated to major activation in the PI (Kirk and Montague, 2015). Interestingly, both studies show a specific modulation in value reward processing in the striatum and vMPFC, from interoceptive body awareness regions (PI) that correspond to bottom-up emotion regulation systems, in line with the bottom-up mechanism hypothesis of emotion regulation changes derived from mindfulness practice.

## Integrating psychological, clinical and neuroscience evidence on emotion regulation in mindfulness research

The field of contemplative science, the scientific study of the effects of mindfulness, and contemplative practices in mental health and biological functions, is fairly new but growing quickly. In this article we have focused exclusively on the relationship between mindfulness practices, using diverse empirical models (dispositional mindfulness, mindfulness inductions, MBIs, and EMs), and emotion regulation functions from psychological and neurobiological perspectives. A range of MBIs have demonstrated utility in several clinical conditions (see Table 1), targeting a myriad of emotion dysregulation symptoms (Gotink et al., 2015).

With the aim of understanding mechanisms underlying mindfulness health benefits, authors have proposed several psychological and neurocognitive models (see Table 2) that cover attention, emotion, and self-awareness systems as target mechanisms (Tang et al., 2015). Here we focused particularly on emotion regulation mechanisms targeted by mindfulness meditation, reviewing different studies using psychological and neuroimaging measurements, ranging from correlational to randomized longitudinal designs.

In the field of mindfulness and emotion regulation, one main claim is that mindfulness might elicit a particular type of emotion regulation strategy often called “mindful emotion regulation” that relies on bottom-up mechanisms, in contrast to cognitive reappraisal, which relies on a top-down mechanism. Although there is no single definition, mindful emotion regulation is conceived as a unique emotion regulation strategy, that results from encountering diverse emotional states from a mindful mental state, which includes awareness and acceptance (Chambers et al., 2009; Farb and Segal, 2012; Chiesa et al., 2013; Grecucci et al., 2015a). In particular, it is stated that bottom-up emotion regulation strategies (like those implied in mindfulness) don't require PFC and top-down mechanisms (Chambers et al., 2009; Farb and Segal, 2012; Chiesa et al., 2013; Grecucci et al., 2015a). In terms of neurobiological emotion regulation systems, these strategies might rely on modification of implicit emotion regulation and emotion generation systems, but not on changes in the explicit emotion regulation system. In this section, in accordance with the reviewed studies, we will assess whether this claim and its assumptions are met.

Studies measuring structural brain changes in EMs highlight changes in the MPFC and diverse subcortical regions, including regions devoted to meta-awareness, memory consolidation, extero-interoception, and emotion regulation (Fox et al., 2014), with no exact matching to bottom-up systems, but with no involvement of typical LPFC. Longitudinal studies with MBIs have also implicated regions typically involved in the same functions described above (like the AI and amygdala), but no changes in the MPFC and LPFC have been found, regions known for top-down emotion regulation. Strikingly, only AI and brainstem regions overlap between EM and MBIs studies, suggesting neuroplasticity in key areas for emotion generation, interoception, mood, and viscerosomatic processing. As mentioned, no inference about causality (in EM studies), nor about brain functions, can be derived from these studies.

Studies measuring dispositional mindfulness have found negative correlations with negative affect and positive correlations with positive affect traits; factorial analysis has pointed out the distinct and interrelated nature of mindfulness and emotion regulation as constructs. Mental health outcomes of mindfulness might be mediated by emotion regulation capacities (Coffey et al., 2010). Similarly, dispositional mindfulness has been linked to a higher right PFC, minor amygdala activation and changes in rs-fMRI in regions from all the emotion regulation systems (see Table 4). These studies provide evidence of top-down regulation mechanisms. As stated early, several limitations preclude an unequivocal interpretation of these findings in the context of mindfulness and emotion regulation research.

Two studies using mindfulness inductions (mindfulness as emotion regulation strategy) have provided preliminary evidence of direct bottom-up regulation engagement, changing the emotion generation system, with no involvement of the PFC. However, these studies lack an alternative cognitive emotion regulation strategy for contrasting the specificity of the strategy (see Table 4). In addition to the noted methodological limitations, we argue that using a unique mindfulness induction session might be insufficient for eliciting a “mindful emotion regulation” strategy and the recruitment of the bottom-up brain systems. Secondly, central to this discussion is the question of how mindfulness as an emotion regulation strategy is defined and operationalized. Is it a formal practice, identical or derived from mindfulness meditation? Or is it a particular state, related to the notion of mindfulness as a transient state? We will return to this discussion in the next section.

Longitudinal studies have yielded mixed results regarding the involvement of different emotion regulation systems (top-down vs. bottom-up). Studies with healthy populations using self-experiential focus recruit emotion-generation (AI, sg-ACC) and body-awareness (AI, SSC) systems. Well-designed RCTs with active control groups have mostly (but not exclusively) demonstrated changes in emotion generation (amygdala, AI, ACC) and implicit emotion regulation systems (v-MPFC, OFC), while being effective in regulating negative emotions. Clinical studies with anxiety disorder populations have shown major involvement of explicit emotion regulation systems (see Table 4). It is worth noting that these differences might be due to methodological limitations (e.g., simple size), but also to the specific cognitive demands of the experimental tasks (such as self-reference, regulation of self-beliefs or affect labeling tasks) that by nature require top-down regulation mechanisms. Overall, changes in bottom-up neural mechanisms are in line with the findings of psychological studies of MBIs, in which decreases in *emotional cognitive reactivity*, and *rumination* strategies, and increases in *mindfulness skills, self-compassion*, and *meta-awareness* emotion regulation strategies, appear to underlie the beneficial effects of MBIs (see Table 3).

Finally, studies with EMs using emotion and pain paradigms have consistently demonstrated changes in bottom-up emotion generation systems (amygdala, AI, sg-ACC), with reported deactivations in, or no involvement of, the PFC. In some studies involving social emotion or reward processing tasks, EMs displayed increased engagement of interoception brain system (mainly PI), modulating emotion generation, and implicit emotion regulation systems of reward-related areas (caudate, putamen, v-MPFC; see Table 4), providing evidence of the engagement of a bottom-up emotion regulation system in EMs.

From the reviewed studies, we argue that there is support for the claim that mindfulness practice changes the bottom-up emotion regulation systems (emotion generation and implicit emotion regulation systems), although this effect diverges across different empirical models dispositional mindfulness, mindfulness inductions, MBIs and EM studies. In line with Chiesa et al. (2013), studies with EMs show a clearer engagement pattern of bottom-up systems, suggesting that these types of strategies are developed through long-term meditation training. However, intervention studies with a RCT design are better suited for providing evidence about a causal relationship between mindfulness training and bottom-up emotion regulation system changes.

### The problem of mindful emotion regulation

From psychological studies, including theoretical and evidence-based psychological models (Table 3), as well as neuroimaging studies (Table 4), it becomes evident that mindfulness (in MBIs and EMs) also engages and requires top-down emotion regulation. As Lutz et al. stated, mindfulness meditation can be conceived as “a family of complex emotional and attentional regulatory strategies developed for various ends” (Lutz et al., 2008b). From a traditional Buddhist psychology perspective, the development and refinement of attention (attention regulation; Grabovac et al., 2011), and the capacity for monitoring and labeling affective states (Analayo, 2003), are central for achieving the intended effects of mindfulness meditation. From this viewpoint, and taking into account models of different emotion regulation brain systems and different emotion regulation strategies, the notion of “mindful emotion regulation” (Chambers et al., 2009; Farb and Segal, 2012; Chiesa et al., 2013; Grecucci et al., 2015a) seems to imply certain problematic aspects.

The notion of “mindful emotion regulation” entails two problematic aspects. The first refers to the nature and definition of the construct “mindful emotion regulation” itself, and the second refers to its brain correlates or engagement/functioning of emotion regulation systems, which we will address separately.

Although we have extensively shown that emotion regulation is (somehow) enhanced by mindfulness practice, we argue that the notion of “mindful emotion regulation” has not been accurately and properly defined. Is “mindful emotion regulation” a psychological trait, stable in time, that diverges across subjects? Or is it a particular mental practice derived from mindfulness? Or is it a mental state, like a transient moment of mindfulness? Generally, the common view across authors is that “mindful emotion regulation” is a *somehow unique* emotion regulation strategy, the result of encountering diverse emotional states from a mindful mental state, including awareness and acceptance (Chambers et al., 2009; Farb and Segal, 2012; Chiesa et al., 2013; Grecucci et al., 2015a). From a first-person perspective, this definition does not make explicit specifications regarding what the practitioner should do while engaging within the emotional state, only succinctly suggesting the gradual development of experiential qualities (attentiveness, acceptance, etc.). What should the focus of attention be (external or internal stimuli)? And, in terms of behavior, what exactly should be done to *perform* the regulation (approach, stop, or hold back)? From a psychological perspective, there is not a clear commitment regarding the unique (or common) involvement of attentional or emotional or body awareness processes. Thus, in line with clinical evidence (Table 3), it is not clear whether “mindful emotion regulation” is properly a unique emotion regulation strategy, with a unique neurocognitive underlying mechanism.

In light of this debate, we argue that “mindful emotion regulation” entails a variety of emotion regulation *processes*, including top-down processes which are cognitively based, involving attention and voluntary cognitive control, conscious monitoring, and explicit regulatory functions; and bottom-up processes, which are affect driven, based on emotion functions that modulates arousal, valence and the encoding of subjective value regarding the triggering stimuli. We argue that “mindful emotion regulation” entails as well a variety of emotion regulation *strategies*, in accordance with the different strategies taught within MBIs and EMs trainings. In this context, we propose a distinction between primarily *top-down* mindfulness-based emotion regulation strategies and *bottom-up* mindfulness-based emotion regulation strategies. Since emotions are multi-componential processes (Thompson, 1990), and like Gross's classification of emotion regulation strategies, our distinction is based on the primary component of the *emotional response* that is targeted and drive the regulation of the emotional state (Koole, 2009).

Top-down mindfulness-based emotion regulation strategies correspond to affect labeling, mindful detachment, dereification, meta-awareness, and cognitive reappraisal, among others, for which *cognitions* and *thought process* are the primary targets of the strategy. Within this group we can find impulses control and emotion dysregulation managing strategies, like those delivered in MBIs (like in DBT and ACT) in which subjects use intentional efforts to increase their attention and awareness capacities for better regulation and control of emotions (Linehan, 1993; Hayes et al., 1999). In this group, dereification and meta-awareness would correspond to more sophisticated strategies, since they involve the development of insight into the nature of the thought process itself (e.g., see thoughts not as facts; Dahl et al., 2015). Using the *process model* of emotion regulation by Gross, we can understand that increases of mindfulness can indeed modulate any of the five stages: selecting or modifying a situation, deployment of attention, changing cognition (cognitive reappraisal), modulating the experience, and behavior, or physiological response (Gross, 2001). This distinction is in line with findings in MBIs (Table 4), and by Chiesa et al. (2013), and is consistent with the claim that novel practitioners in MBIs use primarily top-down emotion regulation strategies.

In bottom-up mindfulness-based emotion regulation strategies, *sensory-perception* and *interoceptive-proprioception* are the primary aspects of the emotional response targeted by the strategies. The bottom-up strategies are characterized by the intentional stance to directly feel (instead of think) or to experience, thus targeting primarily the *feeling* processes (sensory-perception and interoceptive-proprioception). Bottom-up mindfulness-based emotion regulation strategies include concrete experiential explorations that focus for example on unimodal body sensations, like feeling the temperature of the skin, or exteroceptive sensations, feeling the peri-personal space around, to interoceptive sensations, like feeling the internal sensations of the body. Other strategies focus on the broad multi-modal sensory perception of the body, in which interoceptive, exteroceptive sensations, and basic sensory (auditory and visual) perceptions are used as *a whole* as the main focus of intentional experiential explorations (Kabat-Zinn, 2005).

From the above, the bottom-up mindfulness-based emotion regulation strategies range from the titrated exposure to negative sensations (e.g., physical pain), to different body and perception modalities conscious explorations, to the exposure to the complete range of negative and positive emotions without holding or avoiding/rejecting, which are thought within MBIs and EMs trainings. In sum, there is an explicit intention of experiential exploration of bodily sensations (e.g., the *felt sense)* underlying *all* type of emotion and mental content (Hölzel et al., 2011a). For example in the MBCT program, participants are instructed to use the “opening the door of the body” strategy, which invites to be aware of the body sensations that accompany any intense emotions, stepping back from cognitive analysis and rumination and thus cultivating “intimacy” with the raw and usually rejected experience of emotions (Segal et al., 2002). As we have argued, these strategies are primarily the result of changes in bottom-up emotion regulation systems (e.g., exposure to painful feelings), and can be present in mindfulness inductions, MBIs and EMs.

We further noted that studies applying cognitive reappraisal to emotions generated via bottom-up stimulation can result in a paradoxical increase in amygdala reactivity (Herwig et al., 2010; McRae et al., 2012), which in turn can be related to ruminative or repetitive negative thinking as maladaptive cognitive emotion regulation strategies (see Table 3), characteristic of anxiety and depression disorders (Aldao et al., 2010). Dysfunctional top-down emotion regulation in psychiatric conditions such as MDD (Johnstone et al., 2007) might be related to dysfunctional forms of self-evaluative processes such as rumination and worry (Farb and Segal, 2012). In this sense, emotions can be generated from top-down and bottom-up systems (Ochsner et al., 2009; McRae et al., 2012), and the way/pathway emotions are generated seems to play a crucial role in the successfulness of emotion regulation strategies. Bottom-up-generated emotional states, as pain and reward in EM studies reveal, might be best targeted by bottom-up mindfulness emotion regulation strategies (see Table 4).

### Embodied emotions and emotion regulation

Classical theories of emotions from Aristoteles, Spinoza, and Hume have highlighted the importance of the body and physiological aspects of emotions, conceiving them essentially as psychosomatics states (Colombetti and Thompson, 2008). Post Jamesian contemporary authors like Damasio and Prinz assert that emotions are basically the perception of the actual physiological condition, affirming in a broad sense the embodied nature of emotions (Damasio, 1999; Prinz, 2004). As Colombetti et al. noted, cognitivist theories of emotions have neglected the role of the body in the generation of emotional states (Colombetti and Thompson, 2008), and as we argue, as well in the regulation of emotional states.

In this context, one of the problematic aspects of Gross's “process model” of emotion regulation is the assumption of a linear fixed sequence through which emotions are generated, starting from attention to relevant external stimuli, cognitive appraisals, to emotional responses and behaviors as secondarily generated (Koole, 2009). Nevertheless, relevant stimuli can trigger emotions without cognitive reappraisal (e.g., Neumann et al., 2003) and emotions can be generated from the bottom-up systems (Ochsner et al., 2009; McRae et al., 2012). Using magneto-encephalography Rudrauf et al. showed that emotional stimuli elicited early brain activation in the visual cortex, spreading through the ventral visual stream, temporopolar regions, to OFC/vMPFC, ACC, and SSC. This early activation was correlated to arousal ratings and heart beats changes (Rudrauf et al., 2009). Also, it is known that bodily movements can actively influence emotions (Strack et al., 1988; Niedenthal et al., 2005), the manipulation of body posture can alter the regulation of mood (Veenstra et al., 2016), and intentional movement can regulate emotional states (Shafir et al., 2013). From this, even more relevant is the fact that *previous* emotional states can strongly influence cognitions and attention processes (Okon-Singer et al., 2015), which then will drive the emotion regulation process. We argue that this model is fairly reductionist (neurocentric), since it denies the constitutive interwoven nature of body and brain and that their widely known continuous bi-directional interactions are essential for adaptive behavior (Chiel and Beer, 1997).

We argue that the cognitivist “neurocentric” model also disregard the complex reciprocal influences between cortical (high-order) and subcortical (low-order) regions (Okon-Singer et al., 2015). This “corticocentric” model of the brain, in which “high”-order regions dominate “low”-order regions (Parvizi, 2009), fits very well with the “process model” of emotion regulation, in which only the cortical top-down emotion regulation system has a privileged role for regulating emotional states. As we have shown in this article, bottom-up (mindfulness-based) emotion regulation strategies modulate *sensory-perception* and *interoceptive-proprioception* components of the emotional state, due to changes in bottom-up emotion regulation systems. These subcortical systems are central in the homeostatic regulation of neuro-vegetative and visceral functions which provide the *bodily* aspect of emotion experience (Bechara et al., 2000; Critchley et al., 2002).

The enactive approach to mind-brain considers cognition, emotion, and body functions as parts of an integrated system at neurobiological, psychological, and phenomenological domains (Thompson and Stapleton, 2009). One of its central principles is the notion of embodiment, or embodied cognition, which in simple terms claims that the whole body (not only the brain) is involved in building up cognition (Varela et al., 1991; Kiverstein, 2012), and in this particular case the experience of emotions (Colombetti and Thompson, 2008; Slaby et al., 2013; Colombetti, 2014). From this perspective, the emotional or affective dimension is connatural and constitutive of organism's adaptation and agency in the world. Organisms have to be “sensible” to their environment in order to *make sense* and *adaptively respond* to new demands, in this account emotions are inseparable from cognitions (Colombetti, 2014). Central for the affective constitution of organisms, three interrelated activities characterize the embedded body-brain system: the capacity of *self-regulation* of internal states, *sensorimotor coupling* with the environment and *intersubjective interaction* with other agents (Thompson and Varela, 2001).

In this context, we argue that emotions are the *ensuing* and *guiding* state of the organism engagement with the environment (world), in which the regulation of its own internal homeostatic states (humoral, visceral, somatic-motor) is inseparable from the emotional state itself (that is targeted with the regulation). As an example, we cannot think that body temperature (the target of the regulation) is something separate and distinct from the homeostatic mechanisms that continuously regulate body functions to keep the temperature constant (regulation mechanism). In fact, the actual body temperature emerges as the result of the reciprocal interactions of diverse regulatory mechanisms. Derived from this, we propose a preliminary account of emotion regulation as an embodied process, basically rejecting the dualism between emotional states (and its somatic expressions, motor and autonomic systems), and the processes and mechanisms of emotion regulation. Emotions and its experience are the result of the continuous reciprocal interactions of top-down, bottom-up, sensory-perception and interoception processes, in which top-down and bottom-up systems can serve as generative and regulatory mechanisms. As we have reviewed in this paper, both emotion systems participate in the generation and expression of emotional states (Ochsner et al., 2009; McRae et al., 2012), at the same time, both are engaged in the regulation of internal homeostatic states (humoral, visceral) and expressive somatic-motor responses (Frank et al., 2014; Kohn et al., 2014; Etkin et al., 2015).

The embodied approach to emotion regulation regarding the problem of “mindful emotion regulation” allows us to conceive top-down and bottom-up mindfulness based strategies in a dimensional and continuous way. These strategies primarily target different aspects of the emotional state, *cognitions* and *thought process, sensory-perception*, and *interoceptive-proprioception*, and their corresponding neural substrates, in this way, at the same time regulating and *ensuing* the current emotional state. From this, it is possible to understand that even mindfulness induction and MBIs can deploy bottom-up regulation strategies, and also EMs can use top-down emotion regulation strategies as part of their repertoire. At the same time, different mindfulness related practices (as samatha, vipashyana and compassion, etc.), as taught within MBIs and EMs trainings might differentially engage the components of the emotional state (Dahl et al., 2015).

In sum, our approach to emotions and emotion regulation intends to overcome the “neurocentrism” and “corticocentrism” of current cognitivist model of emotion regulation. Our embodied account of emotion regulation considers emotional states and regulatory mechanisms as *inseparable*, relying in shared neural networks. It offers a preliminary new framework for integrating neurobiological, psychophysiological, and psychological systems perspectives on emotion regulation and clinical interventions. It aims to be a multilevel and non-reductive paradigm to advance the understanding of emotion dysregulation psychopathologies and their changes in the context of various biological and psychological treatments.

## Clinical implications: emotion regulation, mindfulness, and psychotherapy

As we have seen, MBIs have shown efficacy in a myriad of psychological disorders, characterized by emotion dysregulation psychopathology (see Table 1). From the perspective of longitudinal, clinical, and affective neuroscience studies, we hypothesize that changes in bottom-up emotion regulation systems might be a key differential feature of MIBs vs. the usual Western psychotherapeutic approaches—more specifically, not in the sense that only MBIs elicit changes in these systems (which is not the case), but in the sense that MBIs explicitly involve the engagement of bottom-up mindfulness emotion regulation strategies, using the *sensory* and *interoceptive* components of emotions as targets and *vehicles* for emotion regulation (according to embodied emotion regulation account).

From a clinical psychotherapeutic perspective, this means that the therapist (or MBI instructor) will be able to guide the patient/client into the application of different top-down and bottom-up mindfulness based strategies. In the case of bottom-up strategies, the clinician encourages the participants to focus on the “bodily” components of different emotional state, always conveying the attitudinal stance of acceptance and openness. In this way, discouraging the intend to control and subjugate negative emotional states, but more importantly, discouraging the use of maladaptive top-down emotion regulation strategies like *avoidance, rumination*, and *suppression* among others.

In this sense, there is a constant incentive to shift from a *self-narrative* perspective (ruminative), based on past or future stories, to a *self-experiential* present-centered perspective, so the experience of emotion is decoupled from maladaptive evaluative cognitions. As stated by Chambers, one main difference between psychotherapeutic interventions like psychoanalysis and CBT, and MBIs, is that the former aim to change the content of emotional states (*self-narratives* and cognitions), while MBIs focus on changing the relationship (and not the content) with the emotional (painful) states (Chambers et al., 2009); changing the perspective from which it is experienced, encouraging acceptance and curiosity about the experience itself (*self-experiential focus)*. From an emotional learning perspective, this process can be seen as an exercise of *exposure* (to certain emotions or experiences), *extinction* of maladaptive cognitions or reactive responses, and *reconsolidation* as a new relationship pattern regarding own experiences or daily life problems (Hölzel et al., 2011a).

### Mindfulness and mentalization in the context of psychotherapy

Mindfulness and mentalization can be conceived as different heuristics and approaches to understand mental health, clinical interventions, and psychopathological developments. The notion of mentalization has a heterogeneous origin, starting from the construct of *theory of mind* developed in the field of etiology/cognitive science (Premack and Wooddruff, 1978), the concept of *symbolization* from psychoanalysis (Choi-Kain and Gunderson, 2008) and the notion of *meta-cognition* from novel developments in the empirical study of attachment (Main, 1991). In clinical terms mentalization is defined as the capacity to understand one's own actions and those of others in terms of intentional mental states like desires, needs, and feelings (Choi-Kain and Gunderson, 2008). According to psychodynamic theories, mentalization is a developmental capacity that depends on the quality of the early mother–infant relationship, the development of secure attachment in the infant and a mother's capacities for mentalization (Fonagy et al., 2002). Originally developed to understand BPD psychopathology, actually its deficit has been implicated in a wide range of conditions including autism and schizophrenia, among others (Roffman et al., 2012). Enhancing mentalization is viewed as a common factor responsible for psychotherapeutic change processes, not only in psychodynamic approaches, but also in other clinical perspectives (Björgvinsson and Hart, 2006 for CBT; Lewis, 2006 for DBT). Moreover, in patients with BPD, increased capacity for mentalization is considered the central mechanism of change in all effective treatments (Fonagy and Bateman, 2006).

Exploring the common ground between mindfulness and mentalization, Goodman (2014) uses four aspects of mentalization: (1) observing mental phenomena, (2) describing or labeling mental phenomena, (3) describing the meaning and motivation of one's own and others' behavior as the product of mental states, and (4) understanding the intrinsic linkage and mutual influence of mental states in oneself and others. Taking into account Baer et al.'s models (see Table 2), Goodman suggests that mentalization and mindfulness overlap in two key areas: observing mental phenomena, and labeling/describing mental phenomena. From the perspective of emotion regulation systems, both mental processes correspond to top-down emotion regulation strategies, such as metacognitive awareness and affect labeling. However, the capacity for attributing intentionality to mental states and for understanding the interpersonal influences of mental states, are distinctive factors of mentalization (Goodman, 2014). Given the interpersonal nature of psychotherapy, mentalization capacities constitute central skills for the therapist (to work with patients) and for the patients (to be developed within the treatment; Fonagy and Bateman, 2006).

Another important difference between mindfulness and mentalization, is the type of relationship intended with mental contents and temporality of life events. As we stated, MBIs don't intend to change mental contents, neither explore life events from the past or future possibilities, its main focus is the present-centered non-evaluative awareness of the self-experience. Unlike mentalization interventions, in which the focus is to explore, cognitively understand and change mental contents, which may be referred to future or past life events, but also to emotions and dysregulated emotional states (Allen, 2006). In line with this, mentalization as an emotion regulation strategy has been considered a top-down strategy, relying in the explicit emotion regulation and in the theory of mind brain systems (Fonagy and Luyten, 2009; Vrticka and Vuilleumier, 2012). As we have stated, MBIs engages bottom-up emotion regulation strategies, which constitutes the distinctive ingredient from other forms of psychotherapies. From our perspective, mindfulness and mentalization have common and different psychobiological functions, which are complementary in the context of treatments for diverse psychopathologies related to emotion dysregulation and mentalization deficits. Nevertheless, further research needs to be done with a view to achieving a better understanding of the biological and psychological differences between these constructs, as well as integrating them properly in psychotherapeutic treatments.

## Conclusions and future directions

Over the last few years, research on contemplative and affective sciences has grown considerably. In this article we have shown how mindfulness is related to emotion regulation using different theoretically and empirically derived models. The main hypothesis explored is that emotion regulation changes are a core mechanism underlying the salutary effects of mindfulness and MBIs. Nevertheless, many of the psychological and neurocognitive theoretical models of mindfulness's mechanisms are not properly and empirically validated. At the same time, empirical studies face many methodological limitations as well.

One important problem is the notion of mindfulness itself. As was mentioned, it has been used for referring to a wide range of psychological phenomena, like a trait (or dispositional mindfulness), a proper meditation practice or a mental state (Davidson, 2010). Even the concept of mindfulness lacks a unique operationalization, since many authors have proposed different definitions, understanding it as an attention capacity, an attitude, a characteristic type of awareness, or even a combination of these (Quaglia et al., 2015). As Grossman states, the complexity of the concept seems more related to a lack of consensus between experts, among other critical issues that constructors of inventories might disregard (Grossman, 2008).

On one side, studies measuring dispositional mindfulness using self-report scales have demonstrated good reliability and convergent validity (Quaglia et al., 2015) and a preliminary coherent putative neural correlate (see Table 4). Coffey et al. have demonstrated that mindfulness and emotion regulation correspond to related but different constructs (Coffey et al., 2010). Nevertheless, the construct of dispositional mindfulness entails several problematic aspects, starting from the assumption that self-report mindfulness scales (basically the self-perception of a person) actually tap into the proper practices of mindfulness (Grossman, 2011). For instance, the specificity of the instruments to MBIs is unknown, e.g., other interventions not based on mindfulness might change the mindfulness level (Lutz et al., 2015). Finally, using these instruments in the context of MBIs might induce biased responses because of the verbal exposure to the word and concept of mindfulness itself, and not because of any actual acquired capacity (Van Dam et al., 2012). Another problematic issue with dispositional mindfulness is the wide range of confounders or variables that actually impact the dispositional “mindfulness level,” including other overlapping and related psychological traits that also vary within normative and clinical populations, like: attention and emotional functions, attitudinal and biased dispositions, prior socialization with the construct and experience with related practices (like yoga or psychotherapy; Quaglia et al., 2015). Future studies will have to control for these factors to better disentangle the nature of dispositional mindfulness as a construct itself.

For longitudinal clinical studies, RCTs with active control groups and multi-arm designs seems to be methodologically the “gold standard” for unraveling the efficacy and effectiveness of a given therapeutic intervention, either for inferiority or superiority studies. As in Zeidan et al. (2015), comparing mindfulness, sham mindfulness, placebo, and control could demonstrate the efficacy of all interventions for pain relief, but noting a differential brain mechanism in emotion regulation of pain (Zeidan et al., 2015). For further understanding the differential engagement of the emotion regulation systems in MBIs, future neuroimaging longitudinal studies will have to explicitly compare different mindfulness instructions within the experimental manipulations (i.e., top-down—attention based vs. bottom-up bodily-based). Then they can explore the acquisition and development of the strategies and their neural correlates. For avoiding problematic aspects of self-report scales, clinical studies should try to include behavioral outcome measures of mindfulness. For better understanding putative mechanisms, longitudinal studies should use several prospective measurements of variables of interest to better disentangle how changes in independent variables and mediators affect dependent variables (Kazdin, 2009).

Using neurobiologically based emotion regulation systems as a framework, we have described how top-down strategies (explicit emotion regulation system) and bottom-up strategies (emotion generation and implicit emotion-regulation systems) can be present within novice and expert meditators. In order to deal with the controversy of emotion regulation mechanisms underlying mindfulness in MBIs and EMs, we have proposed the distinction between mindfulness-based top-down emotion regulation strategies based on attention and acceptance, vs. mindfulness-based bottom-up strategies, which target bodily representations of emotional states. We proposed an *embodied* perspective on emotion regulation as a preliminary framework as a means for understanding different emotion regulation systems, rejecting the dualism between *somatic* emotional states and the processes and mechanisms of emotion regulation. From this, the experience of emotional states is build up from the continuous reciprocal interactions of regulatory mechanisms. This perspective offers an integrative view of cognitive and emotion processes within homeostatic regulatory mechanisms, as well as a non-hierarchical view for conceiving cortical and subcortical systems, as well as brain and body interactions. Further developments might complement this framework integrating first-person phenomenological accounts of emotions and emotion regulation, looking for further integrate experiential and subjective reports with psychophysiological and neurobiological measurements (see Colombetti, 2014, for affective neuro-physiophenomenology).

In line with these recommendations and limitations, from the perspective of methodological and measurement techniques, we suggest that research on mindfulness and emotion regulation should take advantage of mobile device technologies, for example using experience sampling methods, or biological measurements including mobile EEGs or galvanic response devices, thereby increasing the ecological validity of measurements, variables and constructs of interest. Serum biological markers of inflammatory response and neuroplasticity (BDNF, for example) are also of relevance as putative biological mechanisms of MBIs. As regards neuroimaging technologies, future studies might integrate different methods, taking advantage of the specificity of each, for example combining the spatial resolution of MRIs with positron emission tomography (PET), which might help to disentangle differences in neurotransmitters or neuroradiological markers of neuroinflammation. Within MRI techniques, the use of computational modeling might help to build and test more precise and sophisticated theoretical models for understanding cognitive emotional systems underlying mindfulness and emotion regulation. Finally, multivariate pattern analysis is situated at a privileged level for decoding mental states (certain emotion regulation strategies or mindfulness states) from brain signatures using trained classifiers.

Clinical applications of MBIs will require a very good understanding of what's better for whom, and distinguishing what types of psychological treatments, regular psychotherapy (of different types) or MBIs (of different types) are better for different types of depression or anxiety disorder. This leads to another question regarding how to combine different forms of psychotherapy with MBIs in the context of a wider and more comprehensive model of healthcare, even including psychopharmacological treatments. A better understanding of emotion regulation mechanisms underlying mindfulness and psychotherapy, from biological and clinical perspectives, will foster new insights into emotional life and its disturbances, with the purpose of refining and developing better therapeutic interventions for the widespread mental health disorders characterized by emotion dysregulation.

## Author contributions

SG: conceive the original idea of the article, decided the design of each part. Completed the revision of all scientific literature. Performed all draft versions of the document, as well as the final (approval) version to be published. SG is totally accountable for all aspects of the work, and ensures that all different questions regarding any part of the work can be appropriately investigated and resolved. SM: conceive the original idea of the article, decided the design of each part. Completed the revision of all scientific literature. Performed all draft versions of the document, as well as offering important intellectual content for the final version. Also, gave the final approval of the version to be published. HR: conceive the original idea of the article, decided the design of each part. Completed the revision of most scientific literature. Participated in the elaboration of almost all draft versions of the document, as well as offered very important intellectual content for the final version. Also, gave the final approval of the version to be published. HR and SM are totally accountable for all aspects of the work, and ensures that all different questions regarding any part of the work can be appropriately investigated.

## Funding

This review paper was supported by the Fund of the following institutions: CONICYT (National Commission for Scientific and Technological Research, Chile). Beca-Chile Scholarship. Berlin School of Mind and Brain, Humboldt Universität. Fund for Innovation and Competitiveness (FIC) of the Chilean Ministry of Economy, Development and Tourism, through the Millennium Scientific Initiative, Grant N° IS130005.

### Conflict of interest statement

The authors declare that the research was conducted in the absence of any commercial or financial relationships that could be construed as a potential conflict of interest.
